# Analysis of heavy metals in the conversion of lake sediment and restaurant waste by black soldier fly (*Hermetia illucens*)

**DOI:** 10.3389/fbioe.2023.1163057

**Published:** 2023-03-31

**Authors:** Caixi Hu, Longyuan Yang, Hanlin Wang, Xiaopeng Xiao, Zhongwen Wang, Xiangyi Gong, Xianli Liu, Wu Li

**Affiliations:** ^1^ School of Resources and Environmental Engineering, Wuhan University of Science and Technology, Wuhan, China; ^2^ Hubei Key Laboratory of Mine Environmental Pollution Control and Remediation, School of Environmental Science and Engineering, Hubei Polytechnic University, Huangshi, China; ^3^ Changsha Zoomlion Environmental Industry Co., Ltd., Changsha, China; ^4^ Hubei Provincial Key Lab for Quality and Safety of Traditional Chinese Medicine Health Food, Jing Brand Chizhengtang Pharmaceutical Co., Ltd., Huangshi, China

**Keywords:** black soldier fly, lake sediment, restaurant waste, heavy metal, utilization

## Abstract

The risk posed by heavy metals makes it difficult to dispose of sediment contaminants from dredging lakes in China. Black soldier fly (*Hermetia illucens*) can convert organic waste, such as restaurant waste and lake sediment, to high-value-added protein feed and fertilizer. Experimental groups were formed in this study to explore the conversion of heavy metals present in the mixture of restaurant waste and lake sediment by black soldier fly larvae (BSFL). The results demonstrated that BSFL could survive in pure sediment with an 84.76% survival rate. Relative to the substrate, BSFL could accumulate 70-90% zinc (Zn), chromium (Cr), copper (Cu), and 20-40% cadmium (Cd) and lead (Pb). The experimental group 2:3, with 40% lake sediment and 60% restaurant waste, was the best group after conversion for 15 days, which showed a 95.24% survival rate of BFSL, 82.20 mg average weight of BFSL, 8.92 mm average length of BFSL, with varying content of heavy metals such as Cu (43.22 mg/kg), Zn (193.31 mg/kg), Cd (1.58 mg/kg), Cr (25.30 mg/kg) Cr, and Pb (38.59 mg/kg) in BSFL. Furthermore, the conversion residue conforms to the relevant standards of organic fertilizer in China and can be used as organic fertilizer. Overall, the present study shows that black soldier flies can improve the resource utilization of lake sediment, especially by reducing the effect of heavy metals.

## Introduction

Currently, China is in a critical period of urbanization and industrialization. On account of a rapid increase in urban population and the accumulation of environmental pollution, the problem of pollution in urban lakes has become particularly prominent (Qin et al., 2019). Lake pollution not only leads to a serious decline in water quality but also damages the lake ecosystem, thereby affecting the urban landscape and people’s living environment adversely. As an important part of the lake system, lake sediment serves as the carrier, destination, and reservoir for the migration and conversion of several water environmental pollutants in the lake (Huang et al., 2020). The chemical composition of lake sediment is related not only to the urban and industrial layout of the basin but also to the background material in the lake. In the water bodies such as a lake, a constant material exchange occurs between the sediment and the lake. Under certain conditions, some pollutants in the sediment are released again into the water, causing secondary pollution to the lake (Zhu et al., 2018). Several types of methods for lake pollution control are available. Presently, the technologies such as pollution interception, sediment dewatering, sediment masking, chemical sealing, deep aeration, bioremediation, and so on can enter the practical stage to control lake pollution (Zhang et al., 2021). Lake desilting is one of the most effective and direct measures to reduce lake pollution (Yang et al., 2021). However, lake dredging generates a vast quantity of sediments that amass under arbitrary pressure. These sediments occupy not only vast areas but also contain harmful microorganisms along with some heavy metals. Surface runoff may harm the secondary ecosystem if contaminants from sediments enter the soil and surrounding areas (Zhong and Fan., 2007). Rational disposal and comprehensive utilization of lake sediment have become prominent issues in the field of lake pollution control and holistic use of solid waste.

However, the sediment of lake desilting is widely employed in agricultural fertilizers as it contains rich nutrients (Wang et al., 2008). The sediment often contains several heavy metals, especially in mining areas, which seriously limits its resource utilization. Therefore, heavy metal pollution is the main problem related to sediment resource utilization. The objective behind the development of future technology is to provide safe, efficient, and resource-based ecological treatment of solid waste by adopting green and sustainable environmental practices (Zhao et al., 2022).

The black soldier fly (*Hermetia illucens*) (Diptera: Stratiomyidae) can feed on a large variety of rotting organic materials, such as human and animal feces, vegetable and fruit waste, restaurant waste, and digester sludge (Li et al., 2015b; Lalander et al., 2015; Cheng et al., 2017; Mohd-Noor et al., 2017). BSFL can transform low-cost organic waste into high-value biomass, such as protein (32%–58%) and lipids (15%–39%), which can be added to animal, poultry, and fish feed (Makkar et al., 2014; Gold et al., 2018). Elsayed et al. (2022) evaluated bio-recycling of rapeseed straw mixed in different ratios with chicken manure using black soldier fly larvae (BSFL) followed by biodiesel and protein production as an innovative waste management and biorefinery route. Among different treatments, 20% rapeseed straw ratio showed high fiber biodegradation with enhanced larval biomass yield and lipid accumulation. In addition, the efficiency of resource utilization of solid wastes can be improved by using the transformation technology of BSF in combination with other technologies (Li et al., 2015a). Elsayed et al. (2020) studied a new biological refining method, that is, effective conversion of chicken manure and rapeseed straw through anaerobic co-digestion of digestive recovery. The liquid digestion fraction was used for straw pretreatment and the solid fraction was used for feeding the larva of the black soldier fly. The results showed that biogas and biodiesel in the ratio of 1:3 increased the total bioenergy output by 95.7%, 24.6% and 38.7% compared with raw rape straw, pretreatment straw or chicken manure, respectively. The food and Agriculture Organization (FAO) has designated BSFL as one of the resource insects.

In recent years, conversion technology to reduce heavy pollution of organic waste by BSFL has become popular (Eraky et al., 2022). Studies have shown that BSFL could reduce Cd content in pig manure (Wang et al., 2021). BSFL could change the content of available Cu and Zn in pig manure, and the accumulation of Cu and Zn in BSFL after conversion was <10% (Wu et al., 2021). Furthermore, this technology helps evaluate if the toxicity of Cu, Pb, and Zn has any significant impact on the life cycle of BSFL (Diener et al., 2015). This indicates the substantial potential of BSFL to treat lake sediment for fertilizer.

Organic matter in the lake sediment could theoretically be utilized by BSFL. However, the addition of low-value organic waste, such as restaurant waste, could improve the conversion efficiency of BSFL. In the current study, restaurant waste was mixed with varying proportions of lake sediment to make up for the nutrient deficiency of the sediment and boost BSFL growth. The experiments (100% restaurant waste, 1:4, 2:3, 1:1, 3:2, 4:1%, and 100% lake sediment) based on the conversion of lake sediment and restaurant waste by black soldier fly larvae (BSFL) were investigated to explore the accumulation and reduction of typical heavy metals in BSFL and substrate after conversion. Besides, the best experimental group can be found after analyzing the experimental products. In this manner, the findings of this study contribute immensely to the uniform distribution of heavy metals in lake sediment, thereby providing an industrial reference that products can meet the standard.

## Materials and methods

### Raw materials

Black soldier flies (BSF) were established in a greenhouse at Hubei Polytechnic University, China. After hatching, the BSFL were fed with wheat bran for 6 days and then investigated. Restaurant waste (comprising a mixture of breakfast, lunch, and dinner meals left from the previous day) was collected from the Teng long restaurant of Hubei Polytechnic University. Its composition was found to be comparatively stable in the same season. The constitution of restaurant waste is 50% rice, 30% noodles, 10% meat, and 10% vegetables. After manually removing inedible contaminants (e.g., plastic bags, spoons, chopsticks), restaurant waste was crushed into a uniform paste using a pulping machine (ZJJ-550, Jiangsu Kehua Energy Saving and Environmental Protection Equipment Co., Ltd., China). The lake sediment for this study was obtained from five sites in Daye Lake in Huangshi city. Mining pollution causes heavy metal pollution in Daye Lake. The restaurant waste and lake sediment were then stored at 4°C prior to their use. The characteristic features of restaurant waste and lake sediment are shown in Table 1.

**TABLE 1:** Characteristics of restaurant waste and lake sediment.

Characteristics	Restaurant waste	Lake sediment
PH	6.87 ± 0.24	7.35 ± 0.36
Water content/%	27.73 ± 0.49	62.49 ± 2.85
Total organic carbon/%	10.08 ± 0.87	1.35 ± 0.25
Total nitrogen (g/kg)	28.89 ± 3.58	1.80 ± 0.26
Total phosphorus (g/kg)	19.15 ± 1.12	23.52 ± 2.36
Total potassium (g/kg)	3.00 ± 0.36	8.37 ± 0.65

### Experimental design

**FIGURE 1:**
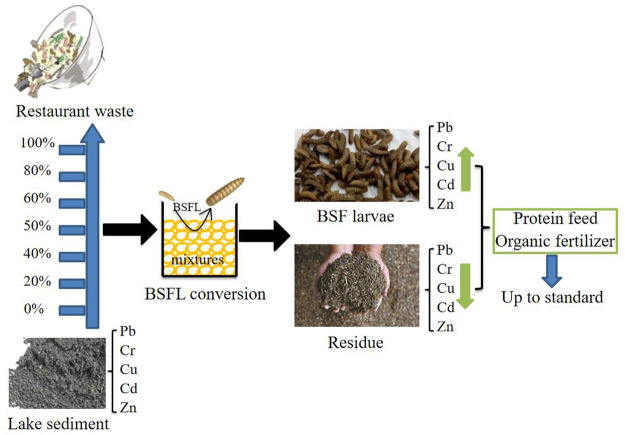
The experimental design of the conversion of lake sediment and restaurant waste by BSFL.

The following five feed mixtures of lake sediment: restaurant waste were formulated: 1:4, 2:3, 1:1, 3:2, 4:1, and two control groups:100% restaurant waste and 100% lake sediment. Each group weighed 500 g and received an injection of 6-day-old larvae (n = 500). Each group was repeated in triplicate, and both the larvae and substrates were incubated in a plastic bucket (Φ = 20 cm, H = 15 cm) with air permeability and a closed greenhouse controlled at about 28°C–30°C. Each bucket was wrapped in plastic with tiny openings to let air through with a view to retaining 60%–80% moisture. The initial heavy metal content of each experimental group has been measured, as shown in Table 2. Samples of 10 larvae and 10 g substrates were collected every 3 days, respectively. The weight and length were determined using electronic balance and Vernier calipers. The average fresh weight and length of each group were measured, and then BSFL was preserved at 4°C temperature. The lake sediment was stored at 4°C after drying. After conversion for 15 days, the experiment was completed. The number of surviving larvae in each group and the survival rates of BSFL were determined. Figure 1 shows the experimental design of the conversion of lake sediment and restaurant waste by BSFL.

**TABLE 2:** Initial content of heavy metals in experiment groups.

	100% RW	1:4	2:3	1:1	3:2	4:1	100% LS
Composition (%)							
Lake sediment	0	20	40	50	60	80	100
Restaurant waste	100	80	60	50	40	20	0
Heavy metals							
Cr/(mg/kg)	19.24 ± 2.47	29.31 ± 0.147	38.47 ± 0.36	44.43 ± 0.87	49.47 ± 2.45	59.54 ± 2.48	69.61 ± 3.98
Zn/(mg/kg)	43.57 ± 3.21	91.64 ± 2.54	141.08 ± 3.54	163.44 ± 1.51	187.77 ± 2.58	235.84 ± 4.59	283.19 ± 5.68
Cd/(mg/kg)	0.37 ± 0.05	0.92 ± 0.09	1.46 ± 0.12	1.73 ± 0.22	2.00 ± 0.18	2.55 ± 0.37	3.09 ± 0.25
Cu/(mg/kg)	15.55 ± 2.06	40.54 ± 0.98	62.78 ± 1.89	72.68 ± 2.65	88.61 ± 1.87	110.49 ± 2.69	133.53 ± 4.68
Pb/(mg/kg)	6.87 ± 1.23	17.05 ± 1.28	28.42 ± 3.25	31.47 ± 2.42	37.58 ± 0.55	47.27 ± 2.59	57.78 ± 5.35

Note: 100% RW-100% restaurant waste, 100% LS-100% lake sediment.

### Heavy metal analysis

The concentration of metals in BSFL was determined, and 8 mL 69% HNO_3_ and 2 mL 30% H_2_O_2_ were added to each 0.50 g sample, then placed into the CEM-MARS 6 S Microwave Digestion System (CEM Corp, Matthews, NC, United States) at 180°C for 15 min. After digestion, the digestion tube was taken out and put on the intelligent temperature control electric heater to dry the acid at 140°C. After acid removal and filtration with water, the volume was fixed to 50 mL, and the heavy metal content was determined using iCAP inductively-coupled plasma mass spectrometer (ICP-MS) (ICP-MS, iCAPQ, Thermo Scientific, United States).

Heavy metal concentration in the substrate was evaluated, and 0.50 g substrate was added with 6 mL 69% HNO_3_, 2 mL 38% HCl, and 2 mL 30% H_2_O_2_ for microwave digestion. The parameters remained unchanged and were above BSFL measurement. After acid removal, the volume was fixed to 50 mL. Heavy metal contents were determined by ICP-MS (ICP-MS, iCAPQ, Thermo Scientific Inc., United States).

### Data analysis

Microsoft Excel 2016 was used to determine the average and standard deviation of data. The figures were drawn with Origin 9.0. Remarkable differences were noticed at the level of 0.05. Required calculations and statistical analysis are furnished below:

The survival rate = W1/(500−W2) ×100%, where W1 is the total number of larvae surviving at the end of the experiment and W2 denotes the total number of larvae sampled per time.

(1) Average length (mm) = Total length of 10 BSFL/10;
(2) Average fresh weight (mg) = Total weight of 10 BSFL/10;
(3) Content of heavy metals in conversion residues = Heavy metal content in conversion residue/Initial heavy metal content in the substrate;
(4) Content of heavy metals in BSFL = 100%—Content of heavy metals in conversion residues.

## Results

### Influence of different substrates on the survival rate and the growth of BSFL

**FIGURE 2:**
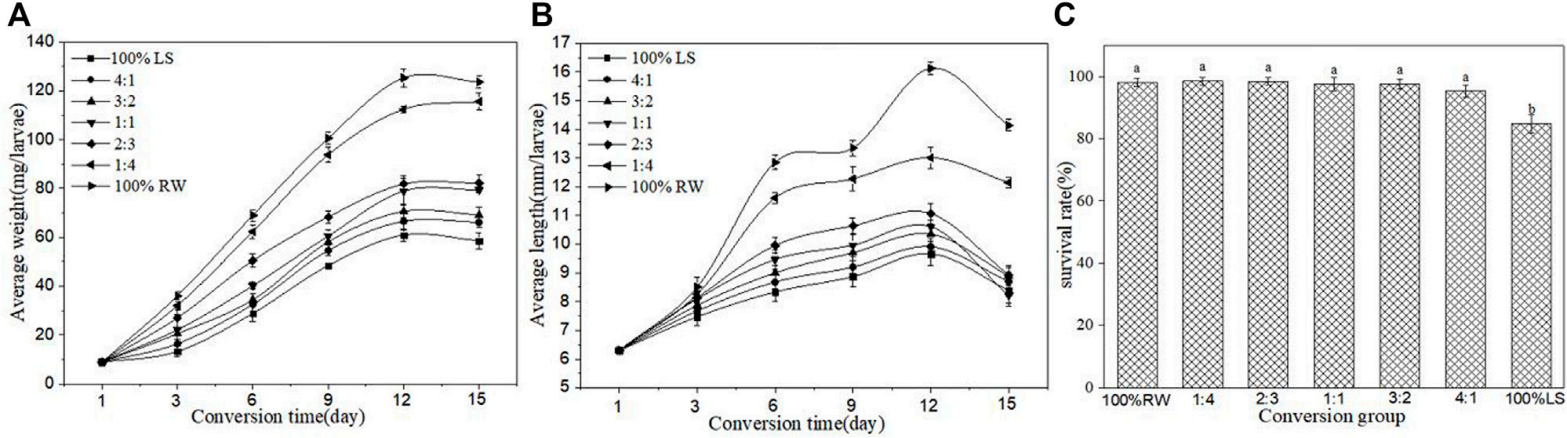
Survival rate and the growth of BSFL during the conversion of different proportions of lake sediment and restaurant waste by BSFL. **(A)** Average weight; **(B)** Average length; **(C)** Survival rate. 100% LS:100% lake sediment, 100% RW:100% restaurant waste. Small letters in the figure indicate no significant difference (n=3, P>0.05).

Figure 2 depicts the survival rate and the growth of BSFL at the time of conversion of lake sediment and restaurant waste by BSFL. It can be concluded that the addition of restaurant waste to sediment could increase the survival rate of BSFL. With the increase in lake sediment in the feeding treatment, BSFL became lighter (Figure 2A) and smaller in size (Figure 2B). The larval weight and length reached their maximum on the 12th day, and thereafter larval length began to decrease. The final weight of larvae in group 100% restaurant waste and 1:4 showed no significant difference, though it was remarkably different from the final weights of other groups (*p* < 0.05). No significant difference was noticed in final larvae weight between 2:3, 1:1, 3:2, 4:1, and 100% lake sediment groups (Figure 2A). The final weight of larvae in group 100% restaurant waste was maximum (123.60 ± 2.50 mg), and the minimum weight of larvae (58.6 ± 3.3 mg) was in group 100% lake sediment. The final length of the group 100% restaurant waste and 1:4 showed a significant difference from that of other groups (*p* < 0.05). No significant difference between groups of 2:3, 1:1, 3:2, 4:1, and 100% lake sediment (Figure 2B) was noticed. The final length was maintained within 8.22–8.92 mm. The group 100% lake sediment had the maximum length (14.14 ± 0.20 mm), while the group 100% restaurant waste had the minimum length (8.22 ± 0.39 mm). Figure 2C shows that the survival rate of BSFL in the control group with pure sediment is the lowest (84.76%). With the increase in restaurant waste, the survival rate of BSFL increased from 95.24% to 98.48%. Each treatment group demonstrated a remarkably significantly higher survival rate of BSFL than the control group with pure sediment. The survival rate increased by 10.48%–13.72% compared with that in the pure sediment group.

### Accumulation of heavy metal Cu in BSFL during the conversion

**FIGURE 3:**
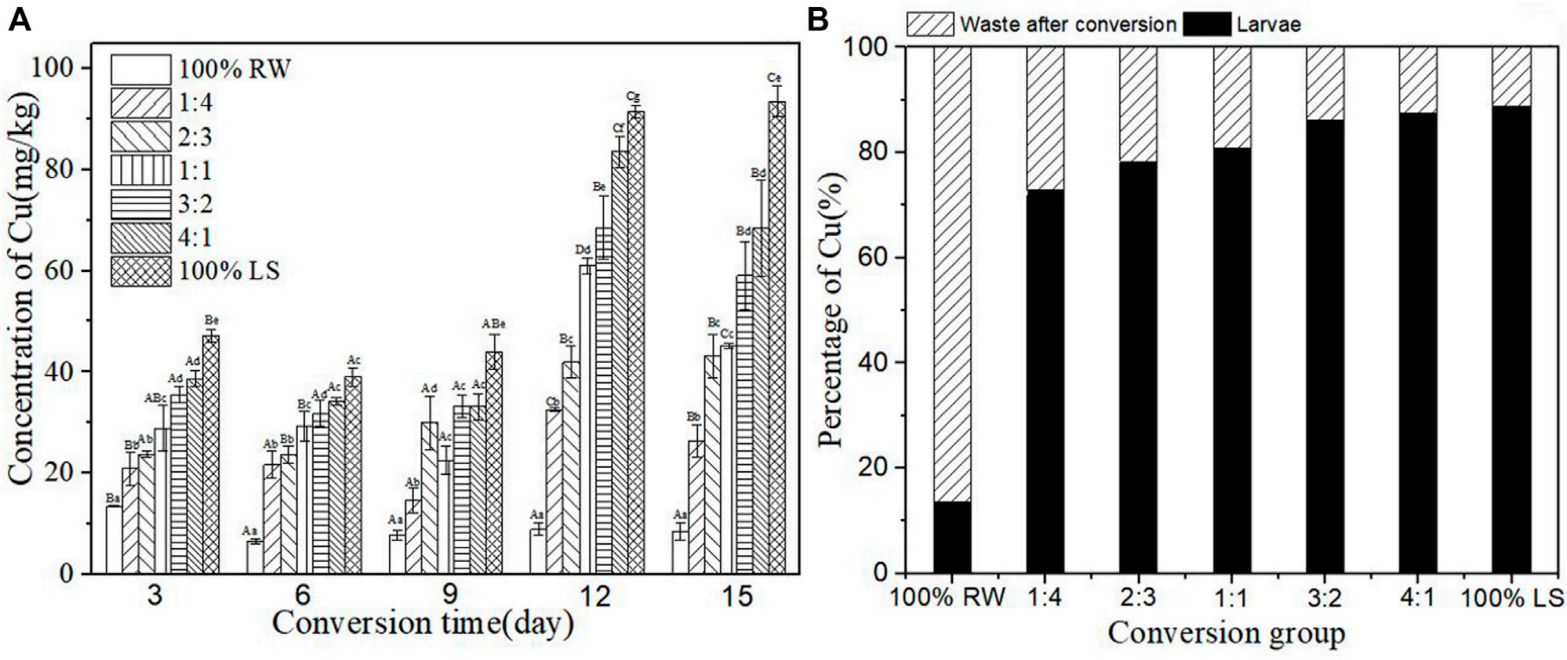
**(A)**-Concentration of heavy metal Cu in BSFL during the conversion; **(B)**: Percentage of Cu in larvae and waste after conversion. 100% LS:100% lake sediment, 100% RW:100% restaurant waste. The small letters in the Figure 3A indicate the significance of heavy metal content in BSFL in different groups at the same time. Capital letters indicate the significance of heavy metal content of the same group at different times (n=3; P > 0.05).

Figure 3A shows the concentration of heavy metal Cu in BSFL during the conversion, which increased with the increase of sediment and revealed a significant difference in each group. The content of Cu in BSFL increased along with the conversion and reached its maximum after 12 days in all groups except the control group, 100% lake sediment. The maximum content of Cu in BSFL in the groups was 83.51 mg/kg. However, this content increased slowly from the 3rd day to the 9th day and increased rapidly from the 9th day to the 12th day. The content of Cu in BSFL in each experimental group increased by 3.11–11.00 times compared with that in the pure restaurant waste group. This suggests that the content of heavy metals in BSFL after conversion could rise by feeding sediment. The percentage of Cu in larvae and waste after conversion is shown in Figure 3B. Compared with the pure restaurant waste group, the content of Cu in BSFL in the experimental group increased by > 70%. This suggests that BSFL could accumulate most of Cu (Figure 3B).

### Accumulation of heavy metal Zn in BSFL during the conversion

**FIGURE 4:**
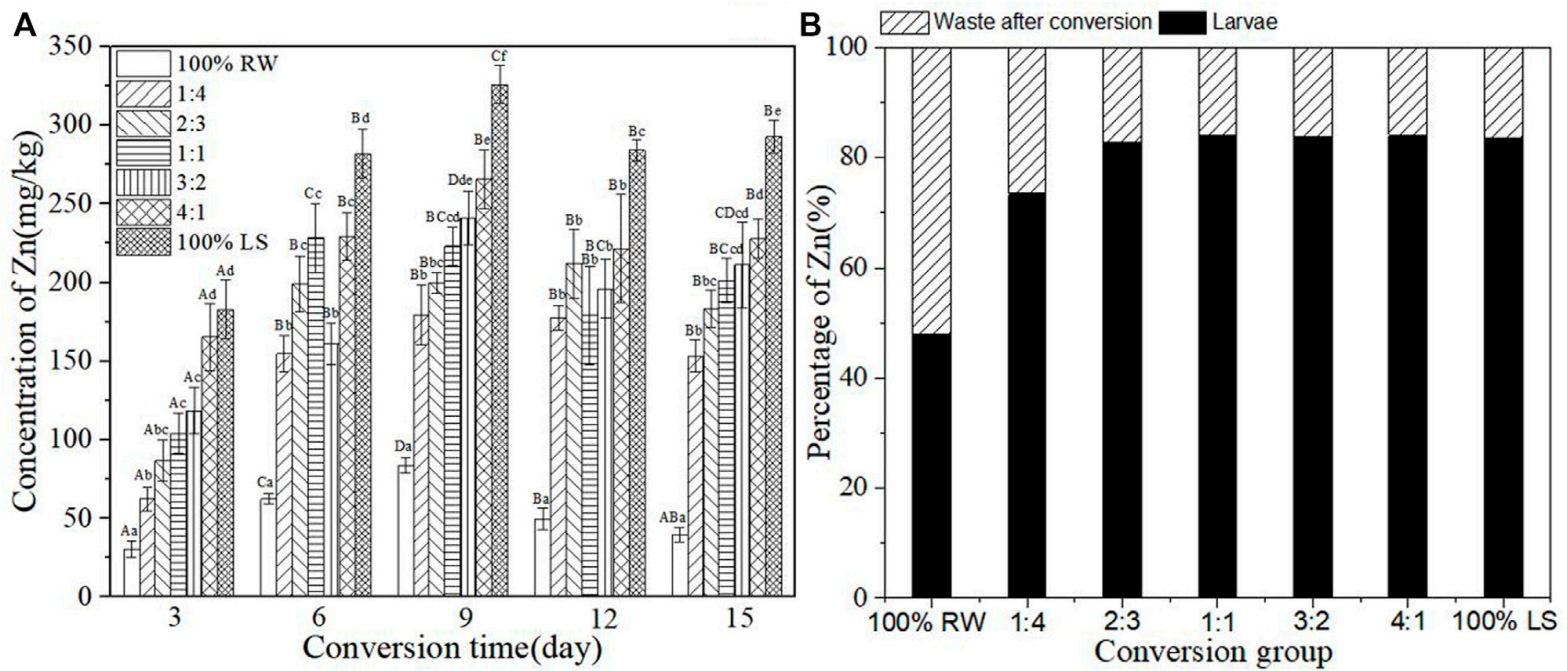
**(A)**-Concentration of heavy metal Zn in BSFL during the conversion; **(B)**-Percentage of Zn in larvae and waste after conversion. 100% LS:100% lake sediment, 100% RW:100% restaurant waste. The small letters in the Figure 4A indicate the significance of heavy metal content in BSFL in different groups at the same time. Capital letters indicate the significance of heavy metal content of the same group at different times (n=3; P > 0.05).

Figure 4A displays the concentration of heavy metal Zn in BSFL during the conversion. The concentration of Zn increased with the increase of sediment. The content of Zn in BSFL increased before 9 days and thereafter decreased. The content of Zn peaked at 325.82 ± 12.03 mg/kg on the 9th day but experienced a slight decrease between the 9th and 15th days. Except for the control group 100% restaurant waste, the content of Zn in BSFL in each group rose by 3.88–7.39 times compared with that in the control group of pure restaurant waste after the conversion. Figure 4B shows the percentage of Zn in larvae and waste after conversion. The experimental groups accumulated >70% Zn, while the control group 100% restaurant waste accumulated 47.79% Zn. This indicates the accumulation of most of the Zn during conversion by BSFL.

### Accumulation of heavy metal Cr in BSFL during the conversion

**FIGURE 5:**
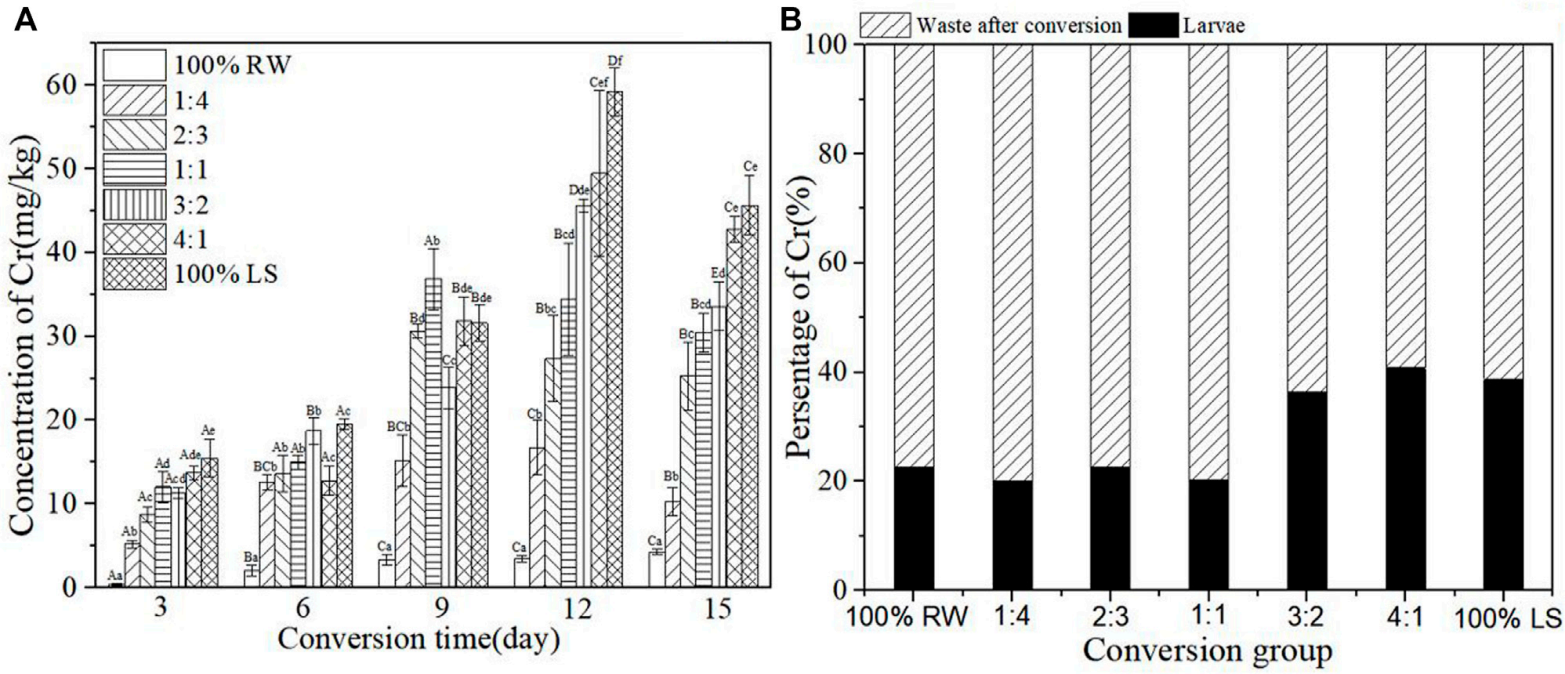
**(A)**: Concentration of heavy metal Cr in BSFL during the conversion; **(B)**: Percentage of Cr in larvae and waste after conversion. 100% LS:100% lake sediment, 100% RW:100% restaurant waste. The small letters in the Figure 5A indicate the significance of heavy metal content in BSFL in different groups at the same time. Capital letters indicate the significance of heavy metal content of the same group at different times (n=3; P > 0.05).

Figure 5A shows the concentration of Cr in BSFL at the time of the conversion process. The concentration of Cr increased with the increase of sediment. The content of Cr in BSFL increased until 12 days and thereafter decreased. The content of Cr in BSFL increased gradually in the first 6 days to about 15 mg/kg. However, the content of Cr in BSFL increased rapidly from day 6–12; the group 100% lake sediment registered the highest increase of 40 mg/kg within 6 days. The content of Cr in BSFL became maximum (42 mg/kg) in group 4:1 on the 12th day. After the conversion, the content of Cr in BSFL in experimental groups increased 2.42–10.74 times compared with the control group of pure restaurant waste. The percentage of Cr in larvae and waste after conversion is shown in Figure 5B. The percentage of Cr in BSFL in group 4:1 was >40% and in other groups <40%, which indicated that BSFL accumulated less and excreted most of Cr into conversion residue. The lower sediment groups (100% restaurant waste, 1:4, 2:3, and 1:1) accumulated about 20% of Cr in BSFL after conversion, while higher sediment groups (3:2, 4:1, and 100% lake sediment) accumulated nearly 40% of Cr in BSFL after conversion.

### Accumulation of heavy metal Pb in BSFL during the conversion

**FIGURE 6:**
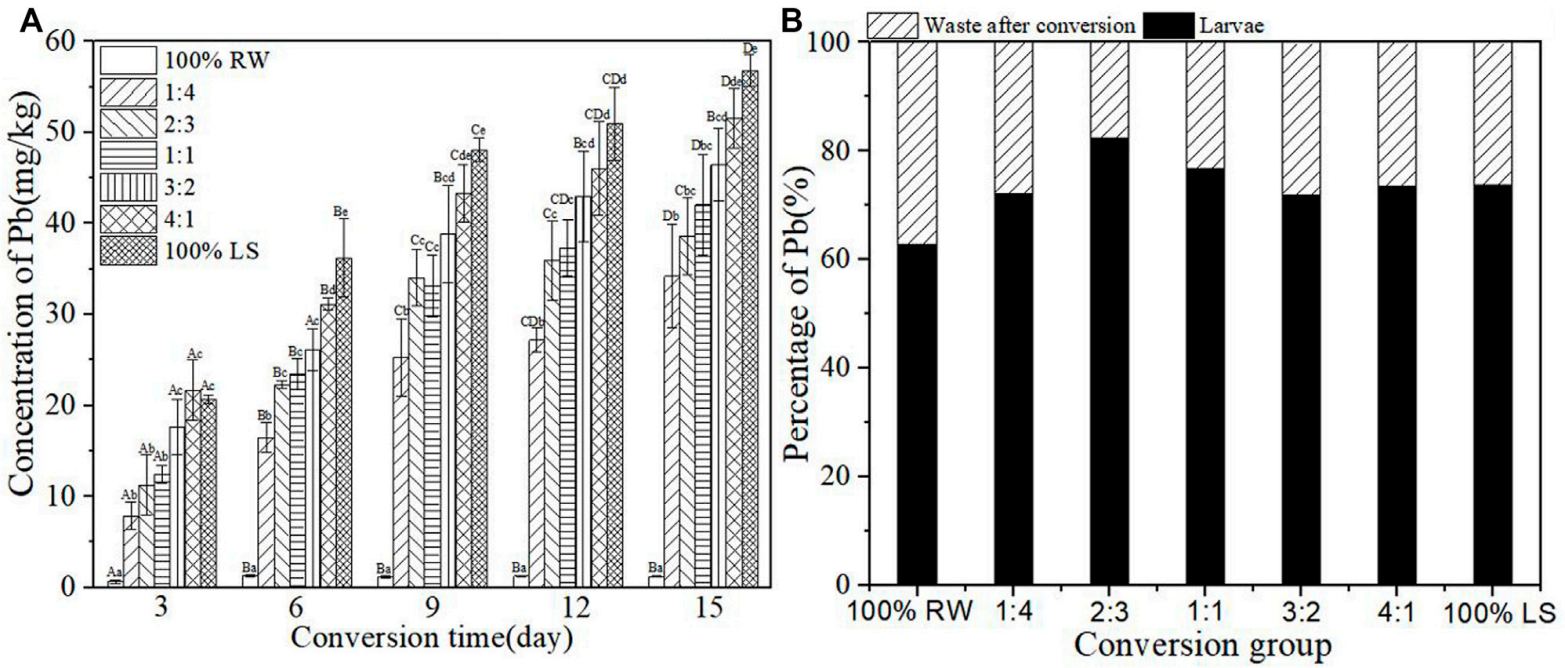
**(A)**-Concentration of heavy metal Pb in BSFL during the conversion; **(B)**-Percentage of Pb in larvae and waste after conversion. 100% LS:100% lake sediment, 100% RW:100% restaurant waste. The small letters in the Figure 6A indicate the significance of heavy metal content in BSFL in different groups at the same time. Capital letters indicate the significance of heavy metal content of the same group at different times (n=3; P > 0.05).

Figure 6A shows the concentration of Pb in BSFL at the time of conversion. The concentration of Pb increased with an increase in lake sediment. The content of Pb increased rapidly in the first 9 days, and the experimental group 4:1 showed the highest content (43.31 mg/kg) of Pb in BSFL. The content of Pb in BSFL in each experimental group rose by 17.80–46.00 times compared with the pure restaurant waste group content but the content of Pb in BSFL accumulated to different degrees. Figure 6B shows the percentage of Pb in larvae and waste after the conversion process. The content of Pb in BSFL in groups was >60%, suggesting the accumulation of most of the Pb in BSFL after conversion.

### Accumulation of heavy metal Cd in BSFL during the conversion

**FIGURE 7:**
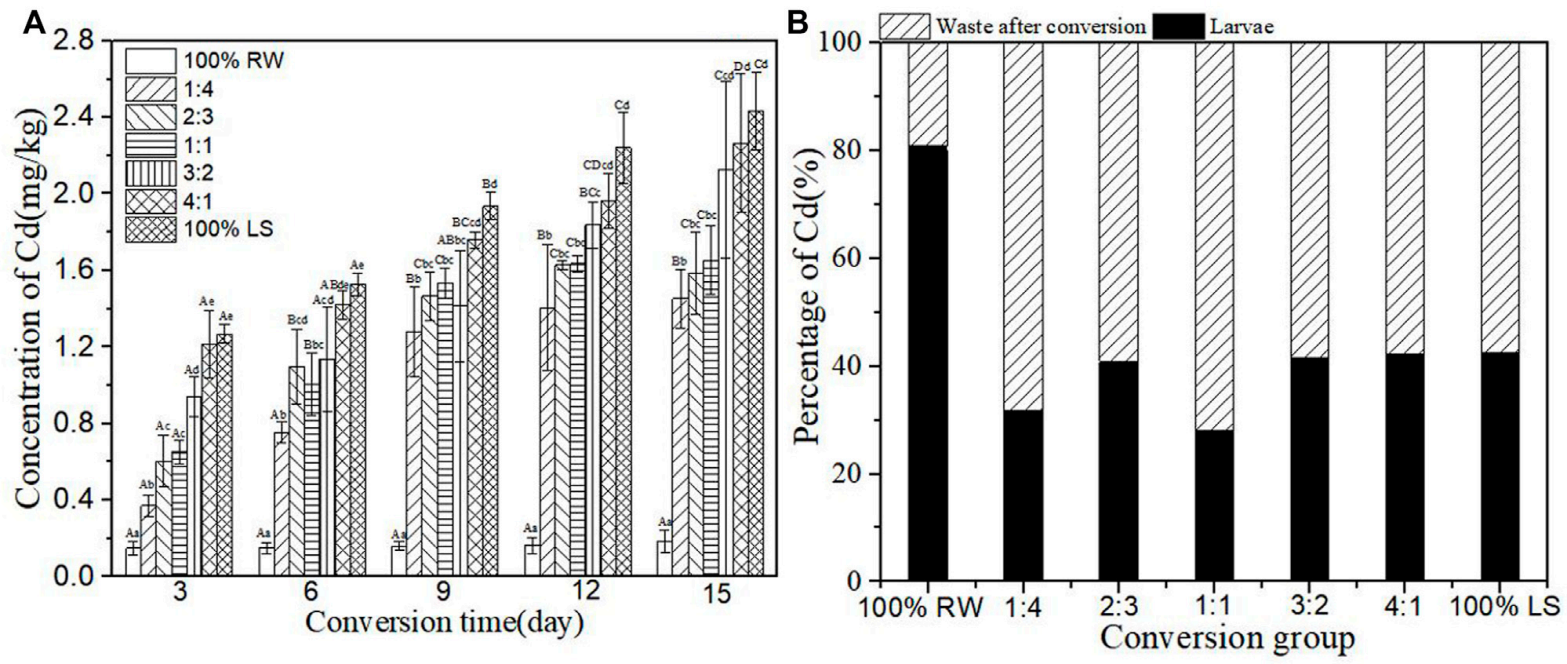
**(A)**-Concentration of heavy metal Cd in BSFL during the conversion; **(B)**-Percentage of Cd in larvae and waste after conversion. 100% LS:100% lake sediment, 100% RW:100% restaurant waste. The small letters in the Figure 7A indicate the significance of heavy metal content in BSFL in different groups at the same time. Capital letters indicate the significance of heavy metal content of the same group at different times (n=3; P > 0.05).

Figure 7A depicts the concentration of Cd in BSFL during the conversion. The concentration of Cd increased with an increase in sediment. The content of Cd increased gradually as the conversion time increased. The content of Cd in BSFL in experimental groups witnessed a rapid increase in the first 9 days. Groups 3:2, 4:1, and 100% lake sediment continued to increase at high speed after conversion for 9 days, but groups 1:4, 2:3, and 1:1 tended to stabilize and did not experience any increase. After the conversion, the content of Cd in BSFL in each experimental group increased by 8.00–15.56 times compared with that in the pure restaurant waste group. The percentage of Cd in larvae and waste after conversion is demonstrated in Figure 7. Except for the group 100% restaurant waste, the percentage of Cd in BSFL in other groups was about 40% after the conversion. This suggested that BSFL accumulated less and excreted most of Cd into conversion residue, or the concentration of Cd may have been influenced by restaurant waste at the time of the conversion by BSFL.

## Discussion

The survival rates of BSFL depend on several factors, such as temperature, humidity, and diet (Tomberlin et al., 2009; Gobbi et al., 2013; Cheng et al., 2017). However, with the increasing application of BSFL in the treatment of organic wastes, several researchers have applied BSFL research for the treatment of organic waste contaminated with heavy metals, such as livestock manure (Wang et al., 2021) and sewage sludge (Cai et al., 2019). Such type of heavy metal-contaminated organic matter may affect the survival rate of the BSFL. Diener et al. (2011) documented that increased dietary Zn content may lead to increased mortality of BSFL. Cd and Cr did not affect the survival of BSFL (Gao et al., 2017). According to the studies, adding water to restaurant waste could increase the survival rate of BSFL. The group of restaurant waste that contained no pure sediment demonstrated the lowest BSFL survival rate, while the group of restaurant waste that had meat sediment showed a higher BSFL survival rate (Figure 2). This corroborates the previous finding by Li et al. (2021). Adding restaurant waste to the substrate for BSFL culture to improve the survival rate of BSFL is not a very rare occurrence and has been used before (Cai et al., 2019). Restaurant waste not only contains dry matter such as protein and fat that can satisfy the growth of BSFL but also sediment that can reduce the moisture of the matrix and improve the pore structure of the matrix, which is conducive to the growth of BSFL. This is consistent with the results of Wei (2020). The survival rate of BSFL in the control group with pure sediment in the present study was significantly lower than in the experimental group supplemented with restaurant waste, which strengthened previous findings by Cai et al. (2019). Different substrates have shown different effects on the growth of BSFL (Meneguz et al., 2018). In this study, the average insect length of BSFL in 100% restaurant waste and 1:4 groups were in the range of 12.15–14.14 mm, and the average insect weight was 115.60–123.60 mg. Both the average insect length and insect weight showed a remarkable difference in 100% restaurant waste and 1:4 groups than in other groups. Li et al. (2021) concluded that adding 50% wheat bran, 30% alfalfa meal, and 20% corn meal resulted in an average insect length of 23.5 mm and an average insect weight of 240 mm, which were significantly different from those observed in the BSFL group fed with other pure mushroom wastes. According to Julita et al. (2018), the proportion of organic waste, such as vegetables, should be increased in the substrate to improve the yield of BSFL. Several studies suggest the best amount of 40% of restaurant waste for addition (Cai et al., 2019). The objective of this study was to use BSFL for the treatment of organic wastewater in lakes to reduce sediment. To achieve this, we must ensure that BSFL operates under the premise of survival and growth in good condition, lower proportion of sediment which content less heavy metal may consistent with the principle.

The accumulation of heavy metals in insects is associated with the concentration of heavy metals present in the diet and the stress time. Some studies have reported that the content of heavy metals in BSFL increased with the increase in treatment concentration (Gao et al., 2017; Wu et al., 2020; Wu et al., 2021). However, in the current study, the content of heavy metals in BSFL increased along with the proportion of lake sediment and the increase of experimental time. The difference was because the content of Zn and Cr reached the highest concentration on the 9th and 12th day, respectively. Further, the accumulation of heavy metals in BSFL increased with the increase of stress generation. Xia et al. (2013) evaluated the accumulation of different concentrations of Zn^2+^ in diets in BSFL at various developmental stages for two consecutive generations and showed a higher concentration of the content of Zn^2+^ in all tissues of the second generation than that in the first generation. Studies have also shown a correlation between the accumulation of heavy metals in BSFL to diet type (Bulak et al., 2020). Cu accumulation of BSFL fed on municipal sludge was found to be higher than that of rainwater sludge (Deng et al., 2022).

BSFL has shown different enrichment mechanisms and tolerance abilities to different heavy metals (Fels-Klerx et al., 2016; Biancarosa et al., 2018). The enrichment mechanism and tolerance ability of BSFL to heavy metals are dependent on the effect of heavy metals on BSFL. Zn is an essential trace element for performing the normal activities of organisms (Davin et al., 2013). Zn is predominantly found in the midgut and epidermal fat in insects (Xia et al., 2013). For the metals in insects to work (Laity and Andrews., 2007), they must bind to the appropriate transcription factor 100% restaurant trash (MTF-1) and absorb Zn autonomically. Zn accumulation varies with the concentration of Zn in a restaurant (Diener et al., 2015). In this study, Zn in BSFL decreased in the later stage, possibly because of a subsequent decrease in its content in the substrate; accumulated Zn in BSFL also decreased through excretion. Cu is mainly found in the cytoplasm of midgut epithelial cells of Dipteran larvae (Southon et al., 2008). When some Cu is accumulated in BSFL, it gets excreted with the growth and metabolism of BSFL; due to this reason, the Cu in BSFL increased first and then decreased in this experiment. The concentration of Cd in BSFL continued to increase during the growing process due to the unique Cd absorption method employed by BSFL and considerably less Cd content in the lake sediment than what the BSFL could tolerate (Gao et al., 2017). The HSP70 protein family can be synthesized in Dipteran cells due to elevated Cd content, protecting other proteins from Cd. This may be the reason for the elevated Cd concentration in BSFL. In this study, Pb accumulation in BSFL increased first and then decreased, supporting the previous findings by Diener et al. (2015). Pb has been found to accumulate significantly in the skin of BSFL (Wang et al., 2018). Resultantly, it can be hypothesized that the mechanism of Pb metabolism in BSFL aims to reduce the Pb content in BSFL through molting. In this study, the concentration of Cr in BSFL increased slowly in the early stage but rapidly later in the middle stage, reached the maximum on the 12th day, and then started decreasing. This may be because of the weak feeding ability of BSFL in the early stage, which leads to the slow accumulation of Cr. In the middle stage, BSFL grow up slightly, and their feeding ability is slightly strengthened, resulting in the rapid accumulation of Cr. The Cr content is slightly reduced subsequently on account of the stress of heavy metals. However, the experiment group of pure lake sediment showed the highest accumulated Cr content (45.67 ± 3.58 mg/kg) in BSFL, in line with the results of Wang et al. (2021).

In the present study, heavy metals in sediment were distributed in BSFL bodies and conversion residues after conversion by BSFL, but the distribution proportions of different heavy metals in BSFL and conversion residues varied (Figure 3). The proportion of Zn, Cu, and Pb in BSFL bodies is higher than in conversion residues, which indicates that BSFL have some enrichment effect on these heavy metals; this finding in the current research further corroborates previous ones by Bulak et al. (2020). The proportion of Zn, Cu, and Pb in BSFL is as high as 70%–90%, which indicates the accumulation of BSFL in most of these heavy metals, and it is inconsistent with findings by Wu et al. (2021). Furthermore, the proportion of Cd and Cr in BSFL is smaller than that in conversion residues, in line with earlier results by Wang et al. (2021).

After the conversion, the BSFL prepupa and the residue can be collected and utilized as animal feed and organic fertilizers. The results of our study were compared with the relevant country (China) regulations on heavy metals in feed in order to evaluate whether the collected prepupa can be used as animal feed. According to the Hygienic Standard for Feed GB 13078- Chinese Code for the Safe Use of Feed Additives (Standardization Administration of the People’s Republic of China, 2017), the maximum amount of Cu and Zn trace elements added is 125 mg/kg and 1,600 mg/kg, respectively. The residual Cu and Zn in BSFL in each experimental group were as per prescribed limits. According to the provisions of China’s Feed Hygiene Standards (Standardization Administration of the People’s Republic of China, 2018), the thresholds of Cr, Cd, and Pb are 200 mg/kg, 2 mg/kg, and 40 mg/kg, respectively, and the content of Cr in BSFL in this study was within the prescribed standard. However, the low sediment contents (experiment groups of 100% restaurant waste, 1:4, 2:3) are safe. However, the experiment group 2:3 with 40% lake sediment and 60% restaurant waste (the best experimental group) could not only meet the standard but also make more use of lake sediment. The residues after the experiment contain nutrient elements (the detailed analysis will be published in further research) that are appropriate for plant growth and can be used as fertilizer. According to China’s Requirements for Fertilizer Classification, the threshold values of Cd, Cr, Cu, Zn, and Pb are 3 mg/kg, 150 mg/kg, 200 mg/kg, 200 mg/kg, and 50 mg/kg, respectively. In the present study, the maximum contents of different heavy metals in residues are 1.95 mg/kg (Zn), 85.70 mg/kg (Cr), 46.67 mg/kg (Zn), 14.98 mg/kg (Cu), 14.07 mg/kg (Pb) (as shown in Table 3). Thus we can conclude that the contents of heavy metals in residues meet the relevant national standards of China, and the residues can be used as organic fertilizer.

**TABLE 3:** Typical heavy metal contents in conversion residues.

	Zn (mg/kg)	Cr (mg/kg)	Cd (mg/kg)	Cu(mg/kg)	Pb(mg/kg)
100% RW	22.75 ± 1.75	18.96 ± 2.78	0.07 ± 0.043	13.45 ± 1.97	3.19 ± 0.76
1:4	24.31 ± 3.89	47.43 ± 3.78	0.81 ± 0.042	11.01 ± 1.67	6.24 ± 0.16
2:3	24.19 ± 3.70	85.70 ± 8.98	1.05 ± 0.024	13.62 ± 1.57	5.01 ± 0.82
1:1	25.95 ± 2.21	68.17 ± 9.73	1.52 ± 0.004	13.96 ± 1.03	9.80 ± 2.00
3:2	30.53 ± 4.16	70.42 ± 2.38	1.35 ± 0.055	12.22 ± 4.84	11.36 ± 2.61
4:1	41.34 ± 5.48	46.84 ± 7.59	1.45 ± 0.028	13.86 ± 1.81	12.35 ± 4.29
100% LS	46.67 ± 2.81	69.77 ± 6.97	1.95 ± 0.114	14.98 ± 1.93	14.07 ± 1.27

Note: 100% RW-100% restaurant waste, 100% LS-100% lake sediment.

## Conclusion

Results of the experiments (100% restaurant waste, 1:4, 2:3, 3:2, 4:1, and 100% lake sediment) based on the conversion of lake sediment and restaurant waste by BSFL reveal that the survival rates of the groups (95.24%–98.48%) were higher than the control group 100% lake sediment (84.76%). A negative correlation was noticed between BSFL and sediment on the average larvae weight and length, and the average larvae weight and length reached a peak on the 12th day. The contents of Cu, Cd, and Pb increased, while the contents of Zn and Cr reached the maximum on the 9th and 12th day, respectively, in the study. Additionally, Zn, Cr, and Cu accumulated 70%–90% in BSFL, while Cd and Pb accumulated 60%–80% in the conversion residues. Safety analysis of products showed that the experiment group 2:3 with 40% lake sediment and 60% restaurant waste was the best group after conversion for 15 days, which had a 95.24% BSFL survival rate, 82.20 mg of average larvae weight, 8.92 mm of average larvae length, and 43.22 mg/kg of Cu, 193.31 mg/kg of Zn, 1.58 mg/kg of Cd, 25.30 mg/kg of Cr, 38.59 mg/kg of Pb in BSFL. Overall, the conversion residue conforms to the relevant standards of organic fertilizer in China and can be employed as organic fertilizer as well. We can conclude that black soldier fly can improve the resource utilization of lake sediment, especially in reducing the effect of some heavy metals. Furthermore, the current study provides a valuable reference for the utilization of lake sediment and the remediation of heavy metal pollution in the mining area in China.

## Data Availability

The original contributions presented in the study are included in the article/Supplementary Material, further inquiries can be directed to the corresponding authors.
